# Associated Factors With Uremic Pruritus in Chronic Hemodialysis Patients: A Single-Center Observational Study

**DOI:** 10.7759/cureus.17559

**Published:** 2021-08-30

**Authors:** Muhammad Sohaib Asghar, FNU Avinash, Manjeet Singh, Muhammad Ali Siddiqui, Syed Adeel Hassan, Shahid Iqbal, Syeda Ghazala Irshad, Mahrukh Zehra, Kainat Siddiqui, Uzma Rasheed

**Affiliations:** 1 Internal Medicine, Dow University Hospital, Karachi, PAK; 2 Medicine and Surgery, Liaquat National Hospital and Medical College, Karachi, PAK; 3 Internal Medicine, Liaquat National Hospital and Medical College, Karachi, PAK; 4 Internal Medicine, University of Louisville, Louisville, USA; 5 Forensic Medicine, Bacha Khan Medical College, Mardan, PAK; 6 Internal Medicine, Dow University of Health Sciences, Dow International Medical College, Karachi, PAK

**Keywords:** dialysis, chronic kidney disease, pruritus, phosphate, potassium, pth

## Abstract

Background and objectives

Uremic pruritus is a recurrent and delicate manifestation in patients suffering from end-stage renal disease. It is a consequence of multiple factors, primarily comprising of metabolic factors and complement activation along with interleukins. The objective of our study was to find out the associated factors of uremic pruritus in chronic hemodialysis patients. The secondary aim was to obtain cut-off values of all the markers predicting pruritus.

Materials and methods

A cross-sectional observational study was conducted in the nephrology department of a tertiary care hospital including 135 patients. The current occurrence of pruritus was diagnosed on the basis of a validated and reliable scale of pruritus among chronic kidney disease (CKD) patients in the local language. Multivariate logistic regression and receiver operating characteristic analysis were conducted to decipher the required objectives.

Results

Study participants had a mean age of 56.29 ± 10.51 years with 56.3% males and 43.7% females. Hypertension was frequent comorbidity (75.6%) followed by diabetes (51.9%). Mean body mass index (BMI), duration of CKD diagnosis, and hemodialysis onset were 26.55 ± 5.37 kg/m^2^, 6.58 ± 3.65 years, and 3.32 ± 2.09 years respectively. Pruritus was reported in 37.0% of the study participants. On multivariate logistic regression, presence of skin allergy (aOR: 8.100 [2.926-22.420], p<0.001), phosphate >4.5 mg/dL (aOR: 3.889 [1.118-15.532], p=0.033), female gender (aOR: 3.592 [1.337-9.655], p=0.011), albumin <3.5 g/dL (aOR: 2.987 [1.156-7.716], p=0.024) and potassium >5.1 mEq/L (aOR: 2.934 [1.030-8.355], p=0.044) were found significantly associated with pruritus.

Conclusion

Many factors were linked to pruritus in hemodialysis patients in the current study. The current study also significantly correlated certain factors with pruritus independently.

## Introduction

Uremic pruritus, also recognized as chronic kidney disease-associated pruritus (CKD-aP), is a recurrent manifestation in patients suffering from end-stage renal disease [1,2]. In the 1970s, around 85% of sufferers of chronic kidney disease developed CKD-aP [1]. Pruritus encountered by patients of chronic kidney disease (CKD) is a consequence of multiple factors, primarily metabolic i.e., hypercalcemia, hyperphosphatemia, hypermagnesemia, secondary hyperparathyroidism, aggregation of itch-inducing mediators such as substance P (a neuropeptide), histamine, and complement activation along with interleukins [2-4]. The quality of CKD-aP varies from patient to patient, being unremitting, immense, and incurable while in many sufferers it can be momentary or restricted to a particular region of the body [5]. In patients with end-stage renal disease, pruritus develops within six months of undergoing hemodialysis [5]. Along with affecting the quality of life and sleep in patients with CKD, pruritus is also recognized as a marker of poor prognosis in hemodialysis patients [2,4]. About 25% of patients suffering from end-stage renal disease develop pruritus during or after undergoing dialysis [4]. Hyperphosphatemia is an extensive complication of end-stage renal disease, if left uncured, can lead to the development of bone pain, pruritus, and clinically deteriorating secondary hyperparathyroidism along with calcification of heart and blood vessels [4,6].

In patients with end-stage renal disease, hyperphosphatemia is a consequence of the underexcretion of phosphorous ions in urine to a level below intestinal absorption [4,5]. Impairment in process of renal phosphate excretion disrupts the control of phosphorous concentration in the body of particularly those patients without residual renal function (RFF) [6]. Dialysis gets rid of one-third concentration of phosphorus while remaining levels are contained by utilization of phosphate binders and dietary restrictions [4,6]. Phosphorus binders inhibit the absorption of phosphorus from the intestines thus regulating the levels of phosphorus in patients with end-stage renal disease [4,6]. Excessive concentration of phosphorus in patients with end-stage renal disease is a marker associated with mortality [6]. Secondary hyperparathyroidism is a prevalent complication endured by patients of end-stage renal disease due to decline in kidney function, abnormality in phosphorus clearance from the body, and inability to bioactivate vitamin D [7]. Cascade of events i.e., increase in extracellular levels of phosphate, declined concentration of ionized calcium in extracellular fluid, significantly decreased calcitriol levels triggers the synthesis of parathyroid hormone (PTH) along with its secretion [6,7]. Extensive secretion of parathyroid hormone leads to hyperplasia of the gland, thus with the advancement of the disease, downregulation of vitamin D receptors (VDR) and calcium-sensing receptors (CaR) occurs resulting in impairment of control of parathyroid cell function in renal disease patients [8]. Disruption in parathyroid hormone levels (PTH) in patients of chronic kidney disease results in multifaceted complications i.e., pruritus, renal osteodystrophy, neuropathy, cardiac disorders, and vascular calcification, rendering it as uremic toxin [7,8]. Interventions, in order to curtail the toxic effects of hyperparathyroidism, comprise of modification by dietary intake of calcium and phosphorus, dialysis, active vitamin D compounds intake along with analogs, phosphate binders, calcimimetics, and bisphosphonates. Parathyroidectomy is opted as a surgical intervention in case of worsening condition or severe secondary hyperparathyroidism [7,8].

The objective of the current study was to find out the associated factors of uremic pruritus in chronic hemodialysis patients. The secondary aim was to obtain cut-off values for all the markers predicting pruritus. There isn't any data published similar to the present study's objective of obtaining cut-offs, hence it justifies the importance of this analysis.

## Materials and methods

A cross-sectional observational, descriptive study was conducted in the nephrology department of a tertiary care hospital having one of the largest dialysis units in the city. Above 200 patients are enrolled in this dialysis center for regular dialysis. A sample size of 132 was calculated by using a Rao-soft sample size calculator [(Raosoft Inc., Seattle, WA) (http://www.raosoft.com/samplesize.html)], with a population size of 200, a response distribution of 50%, and a confidence interval of 95%. Ethical approval was taken in this study from the institutional review board of Dow University Hospital (IRB/DUH/2021/742/021), and consent to participate has been taken from all the participants.

**Figure 1:**
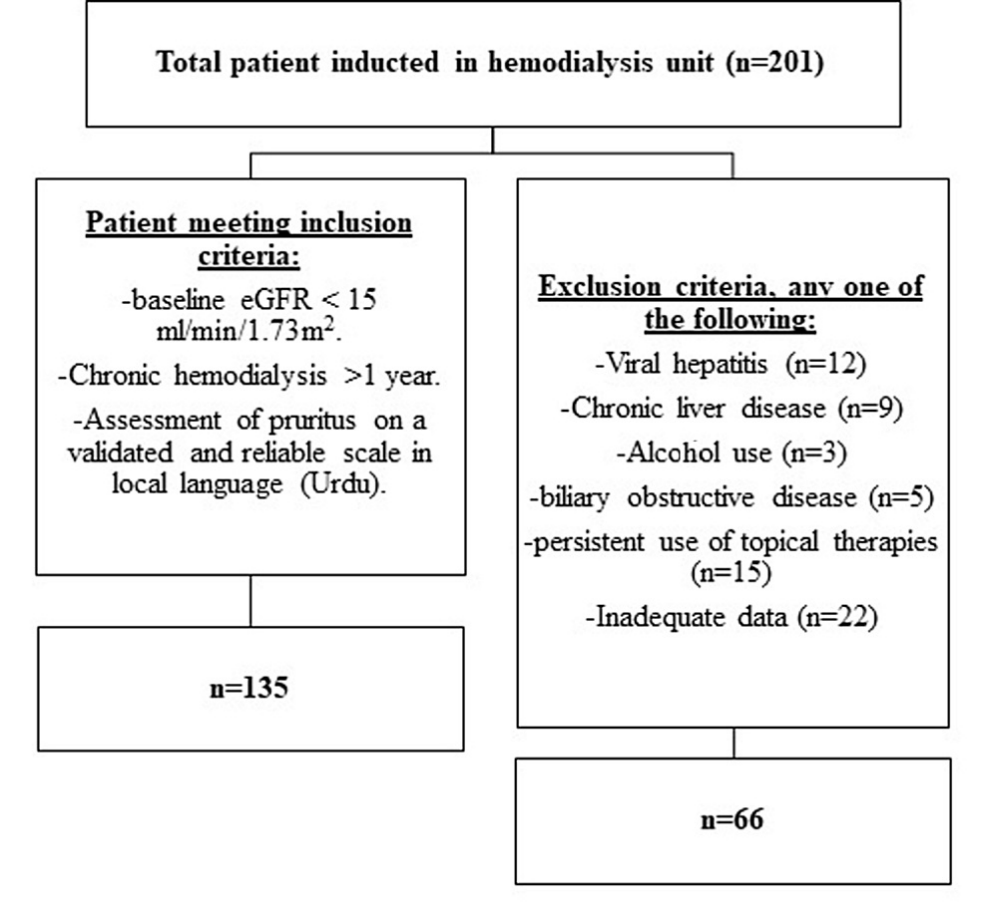
Methodology flow diagram for inclusion and exclusion criteria. eGFR: estimated glomerular filtration rate; n: number of subjects.

A proforma was designed having two sections; the first section covered demographic details including name (as optional), age (in years), gender, body mass index (BMI), co-morbidities including hypertension, diabetes, status of hemodialysis, and duration of CKD. While the second section includes the laboratory markers (including serum urea, creatinine, PTH, potassium, albumin, uric acid, and calcium). These laboratory markers were recorded by a retrospective chart review with a maximum limit of one month prior to the recruitment. The study excluded all the patients diagnosed with viral hepatitis, chronic liver disease, alcohol use, or any other liver morbidity (biliary obstructive diseases), persistent use of topical therapies other than steroids or calamine lotion, as shown in Figure 1.

The study participants were included via a non-probability consecutive sampling technique. The criteria for inclusion were baseline estimated glomerular filtration rate (eGFR) below 15 ml/min/1.73m^2^ and been on hemodialysis for at least one year. Patients on peritoneal dialysis were excluded. The current occurrence of pruritus was diagnosed on the basis of a validated and reliable scale of pruritus among CKD patients in the local language (Urdu) [9].

After collection, the data was analyzed using SPSS Statistics version 25.0 (IBM Corp., Armonk, NY), and results were obtained. The descriptive figures were presented as mean and standard deviation (SD), categorical data were presented as frequency (percentages). Normality of data was determined by Kolmogorov-Smirnov test. Univariate logistic regression was applied to all the study variables with pruritus as dependent variable and unadjusted crude odds ratio (OR) were obtained, further only significant factors were adjusted for a multivariate regression model to obtain adjusted odds ratio (aOR) along with 95% confidence interval (considered significant if p-value <0.05 {two-tailed}). A receiver operating characteristic (ROC) analysis was performed to obtain cut-off levels of the biomarkers associated with pruritus. Laboratory values were also categorized by 2x2 contingency tables to obtain appropriate sensitivity, specificity, positive predictive value (PPV), and negative predictive value (NPV).

## Results

A total of 135 participants met the inclusion criteria with a mean age of 56.29 ± 10.51 years with 56.3% males and 43.7% females. Hypertension was the most frequent comorbidity (75.6%) followed by diabetes (51.9%). Mean BMI, duration of CKD diagnosis, and hemodialysis onset were 26.55 ± 5.37 kg/m^2^, 6.58 ± 3.65 years, and 3.32 ± 2.09 years respectively. Pruritus was reported in 37.0% of the study participants, among whom suffered on daily basis (25.2%), once weekly (7.4%), and once monthly (4.4%). Study variables included a previous history of skin allergy (30.4%), use of calamine lotion (37.7%), topical steroids (10.4%), erythropoietin (60.0%), connective tissue disorder (3.7%), thrice-weekly dialysis (51.9%) and twice-weekly dialysis frequency (48.1%). The mean levels of various biochemical markers and their deranged frequencies are given in Table 1.

**Table 1:** Baseline data of the study population (n=135). Qualitative descriptives are presented as mean & standard deviation (± SD). Quantitative data are presented as frequency % percentages (%). CKD: chronic kidney disease; BMI: body mass index; n: number of subjects; GFR: glomerular filtration rate.

Variables	Descriptives/Frequency
Mean age (in years)	56.29 ± 10.51
Age>50 years	n=90 (66.7%)
Female gender	n=59 (43.7%)
Diabetes	n=70 (51.9%)
Hypertension	n=102 (75.6%)
Body mass index (kg/m^2^)	26.55 ± 5.37
BMI >26.0 kg/m^2^	n=74 (54.8%)
Duration of CKD diagnosis (in years)	6.58 ± 3.65
Duration of hemodialysis onset (in years)	3.32 ± 2.09
Mean estimated GFR ml/min/1.73m^2^	13.76 ± 1.61
Phosphate (mg/dL)	5.56 ± 2.29
Phosphate >4.5 mg/dL	n=83 (61.5%)
Parathyroid hormone (pg/mL)	598.17 ± 463.29
Parathyroid hormone >300 pg/mL	n=101 (74.8%)
Albumin (g/L)	3.55 ± 0.50
Albumin <3.5 g/L	n=62 (45.9%)
Calcium (mg/dL)	8.22 ± 0.88
Calcium <8.5 mg/dL	n=99 (73.3%)
Uric acid (mg/dL)	6.44 ± 1.09
Uric acid >6.5 mg/dL	n=71 (52.6%)
Potassium (mEq/L)	5.01 ± 1.00
Potassium >5.1 mEq/L	n=55 (40.7%)
Urea (mg/dL)	106.74 ± 39.40
Urea >100 mg/dL	n=84 (62.2%)
Creatinine (mg/dL)	7.89 ± 2.32
Creatinine >6.0 mg/dL	n=109 (80.7%)
History of skin allergy	n=41 (30.4%)
Use of calamine lotion	n=51 (37.7%)
Use of erythropoietin	n=81 (60.0%)
Use of topical steroids	n=14 (10.4%)
Use of calamine lotion	n=36 (26.6%)
Connective tissue disorder	n=5 (3.7%)
Twice weekly hemodialysis	n=65 (48.1%)
Thrice weekly hemodialysis	n=70 (51.9%)
Pruritus absent	n=85 (63.0%)
Pruritus present	n=50 (37.0%)
Frequency of pruritus: monthly	n=6 (4.4%)
Frequency of pruritus: weekly	n=10 (7.4%)
Frequency of pruritus: daily	n=18 (13.3%)
Frequency of pruritus: multiple times a day	n=16 (11.9%)

On univariate regression model, factors found associated with pruritus were age >50 years (OR: 2.800 [1.237-6.336], p=0.013), female gender (OR: 2.532 [1.237-5.183], p=0.011), phosphate >4.5 mg/dL (OR: 3.556 [1.576-8.020], p=0.002), albumin <3.5 g/dL (OR: 4.278 [2.019-9.064], p<0.001), potassium >5.1 mEq/L (OR: 4.875 [2.289-10.381], p<0.001), presence of skin allergy (OR: 8.401 [3.665-19.257], p<0.001), and twice weekly dialysis (OR: 2.143 [1.052-4.367], p=0.036). On multivariate logistic regression, female gender (aOR: 3.592 [1.337-9.655], p=0.011), phosphate >4.5 mg/dL (aOR: 3.889 [1.118-15.532], p=0.033), albumin <3.5 g/dL (aOR: 2.987 [1.156-7.716], p=0.024), potassium >5.1 mEq/L (aOR: 2.934 [1.030-8.355], p=0.044), and presence of skin allergy (aOR: 8.100 [2.926-22.420], p<0.001) were independent factors associated with pruritus in hemodialysis patients as shown in Table 2.

**Table 2:** Univariate and Multivariate logistic regression model of study variables for pruritus. * signifies p-value less than 0.05, ** signifies p-value less than 0.01, ^†^ signifies p-value less than 0.001. EPO: erythropoietin; BMI: body mass index; PTH: parathyroid hormone.

Variables	Odds ratio	95% confidence interval		p-value	Adjusted odds ratio	95% confidence interval		p-value
		Lower	upper			lower	upper	
Age <50 years	1.000	-	-	-	1.000	-	-	-
Age >50 years	2.800	1.237	6.336	0.013*	1.571	0.523	4.716	0.421
Gender (Male)	1.000	-	-	-	1.000	-	-	-
Gender (Female)	2.532	1.237	5.183	0.011*	3.592	1.337	9.655	0.011*
Diabetes (Absent)	1.000	-	-	-	1.000	-	-	-
Diabetes (Present)	1.688	0.831	3.426	0.148	-	-	-	-
Hypertension (Absent)	1.000	-	-	-	1.000	-	-	-
Hypertension (Present)	1.039	0.460	2.347	0.927	-	-	-	-
BMI <26 kg/m^2^	1.000	-	-	-	1.000	-	-	-
BMI >26 kg/m^2^	1.333	0.657	2.707	0.426	-	-	-	-
Phosphate <4.5 mg/dL	1.000	-	-	-	1.000	-	-	-
Phosphate >4.5 mg/dL	3.556	1.576	8.020	0.002^**^	3.889	1.118	13.532	0.033*
PTH <300 pg/mL	1.000	-	-	-	1.000	-		-
PTH >300 pg/mL	0.857	0.352	2.086	0.734	-	-		-
Albumin >3.5g/L	1.000	-	-	-	1.000	-		-
Albumin <3.5 g/L	4.278	2.019	9.064	<0.001^†^	2.987	1.156	7.716	0.024*
Calcium >8.5 mg/dL	1.000	-	-	-	1.000	-	-	-
Calcium <8.5 mg/dL	1.667	0.723	3.843	0.231	-	-	-	-
Uric acid >6.5 mg/dL	1.000	-	-	-	1.000	-	-	-
Uric acid <6.5 mg/dL	0.848	0.421	1.706	0.644	-	-	-	-
Potassium <5.1 mEq/L	1.000	-	-	-	1.000	-		-
Potassium >5.1 mEq/L	4.875	2.289	10.381	<0.001^†^	2.934	1.030	8.355	0.044*
Urea <100 mg/dL	1.000	-	-	-	1.000	-	-	-
Urea >100 mg/dL	2.074	0.990	4.346	0.053	-	-	-	-
Creatinine <6.0 mg/dL	1.000	-	-	-	1.000	-	-	-
Creatinine >6.0 mg/dL	2.769	0.968	7.922	0.058	-	-	-	-
History of skin allergy (Absent)	1.000	-	-	-	1.000	-	-	-
History of skin allergy (Present)	8.401	3.665	19.257	<0.001^†^	8.100	2.926	22.420	<0.001^†^
Use of EPO (No)	1.000	-	-	-	1.000	-	-	-
Use of EPO (Yes)	1.978	0.944	4.147	0.071	-	-	-	-
Use of steroids (No)	1.000	-	-	-	1.000	-	-	-
Use of steroids (Yes)	2.508	0.816	7.707	0.108	-	-	-	-
Use of calamine (No)	1.000	-	-	-	1.000	-	-	-
Use of calamine (Yes)	0.583	0.278	1.225	0.155	-	-	-	-
Connective tissue disorder (Absent)	1.000	-	-	-	1.000	-	-	-
Connective tissue disorder (Present)	2.649	0.427	16.425	0.295	-	-	-	-
Mode of dialysis (Thrice weekly)	1.000	-	-	-	1.000	-	-	-
Mode of dialysis (Twice weekly)	2.143	1.052	4.367	0.036*	1.567	0.563	4.359	0.389

**Figure 2:**
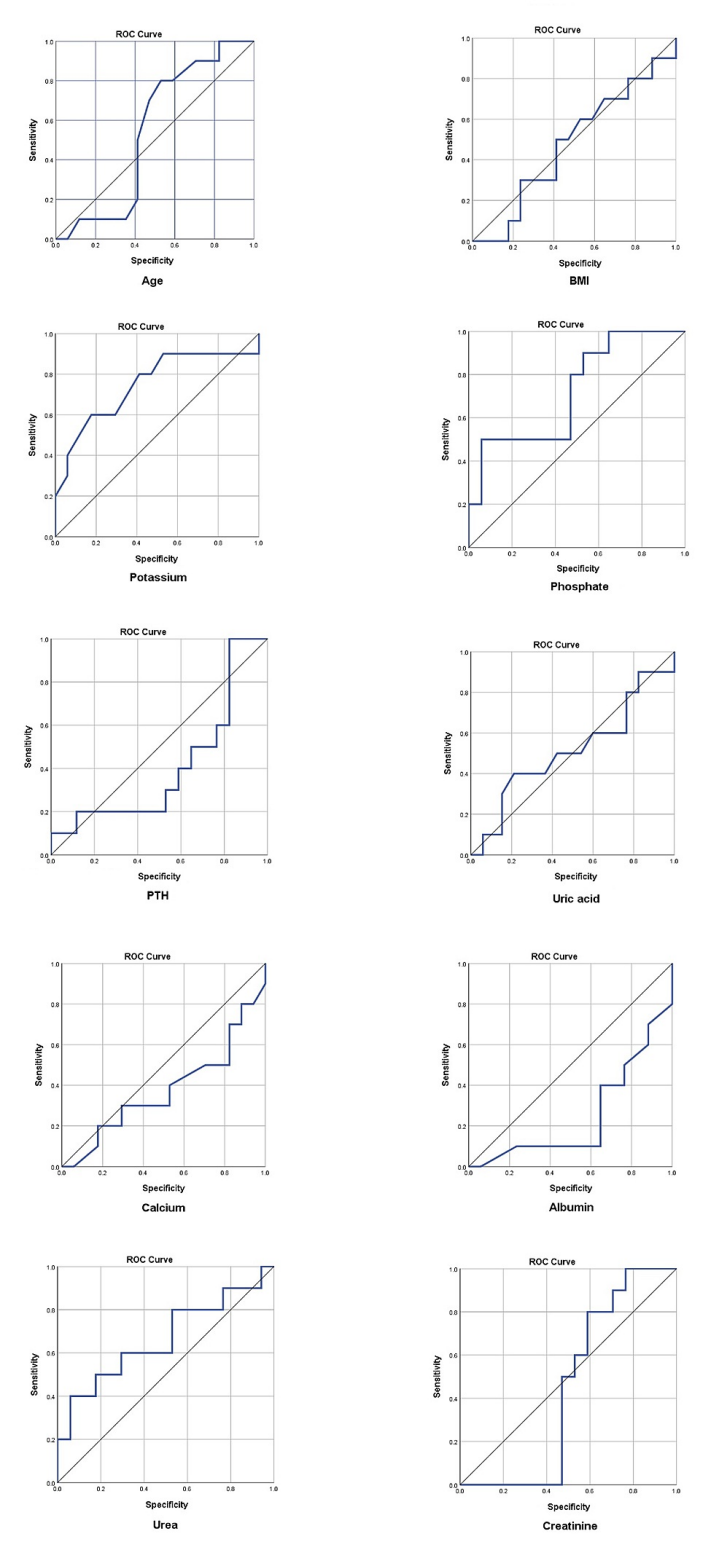
Showing ROC curves for each individual study variable for predicting pruritus. ROC: receiver operating characteristic; PTH: parathyroid hormone; BMI: body mass index.

ROC analysis showed serum potassium at a cut-off value 4.85 mEq/L with area under the curve (AUC) of 0.747, 80.0% sensitivity and 83.3% PPV is predictive of pruritus (p<0.001). Serum calcium at a cut-off value 7.65 mg/dL (AUC: 0.376) with sensitivity of 70.0% and NPV of 50.0% (p=0.017), serum albumin at a cut-off value 3.05 g/dL (AUC: 0.250) with sensitivity of 70.0% and PPV of 40.0% (p<0.001), serum phosphate at a cut-off value 4.45 mg/dL (AUC: 0.724) with sensitivity of 90.0% and PPV of 88.9% (p<0.001), and serum urea at a cut-off value 135.88 mg/dL (AUC: 0.665) with specificity of 94.1% and PPV of 72.7% (p=0.001) are predictive of pruritus presence in hemodialysis patients as shown in Table 3 and Figure 2.

**Table 3:** ROC analysis of descriptive variables for pruritus. * indicates a significant p-value of less than 0.05. ROC: receiver operating characteristics; AUC: area under the curve; S.E: standard error; SEN: sensitivity; SPE: specificity; PPV: positive predictive value; NPV: negative predictive value; PTH: parathyroid hormone; BMI: body mass index.

Variable	AUC	S.E	95% Confidence interval		SEN	SPE	NPV	PPV	Youden’s index	p-value
			lower	upper						
Age (cut-off: 54.0 years)	0.544	0.050	0.446	0.642	80.0%	47.1%	47.1%	80.0%	0.271	0.393
BMI (cut-off: 26.65 kg/m^2^)	0.476	0.051	0.376	0.577	50.0%	58.8%	41.7%	66.7%	0.088	0.649
Calcium (cut-off: 7.65 mg/dL)	0.376	0.052	0.275	0.478	70.0%	17.6%	50.0%	33.3%	0.008	0.017*
Albumin (cut-off: 3.05 g/dL)	0.250	0.044	0.164	0.336	70.0%	11.8%	31.8%	40.0%	0.008	<0.001*
Uric acid (cut-off: 7.10 mg/dL)	0.514	0.054	0.408	0.619	40.0%	78.8%	52.6%	69.1%	0.018	0.793
Potassium (cut-off: 4.85 mEq/L)	0.747	0.047	0.654	0.840	80.0%	58.8%)	53.3%	83.3%	0.388	<0.001*
Phosphate (cut-off: 4.45 mg/dL)	0.724	0.045	0.635	0.812	90.0%	47.1%	50.0%	88.9%	0.371	<0.001*
PTH (cut-off: 134.50 pg/mL)	0.406	0.053	0.303	0.509	90.0%	17.6%	39.1%	75.0%	0.076	0.068
Urea (cut-off: 135.88 mg/dL)	0.665	0.051	0.564	0.765	40.0%	94.1%	80.0%	72.7%	0.341	0.001*
Creatinine (cut-off: 6.80 mg/dL)	0.447	0.050	0.349	0.545	80.0%	41.2%	44.4%	77.8%	0.212	0.305

**Figure 3:**
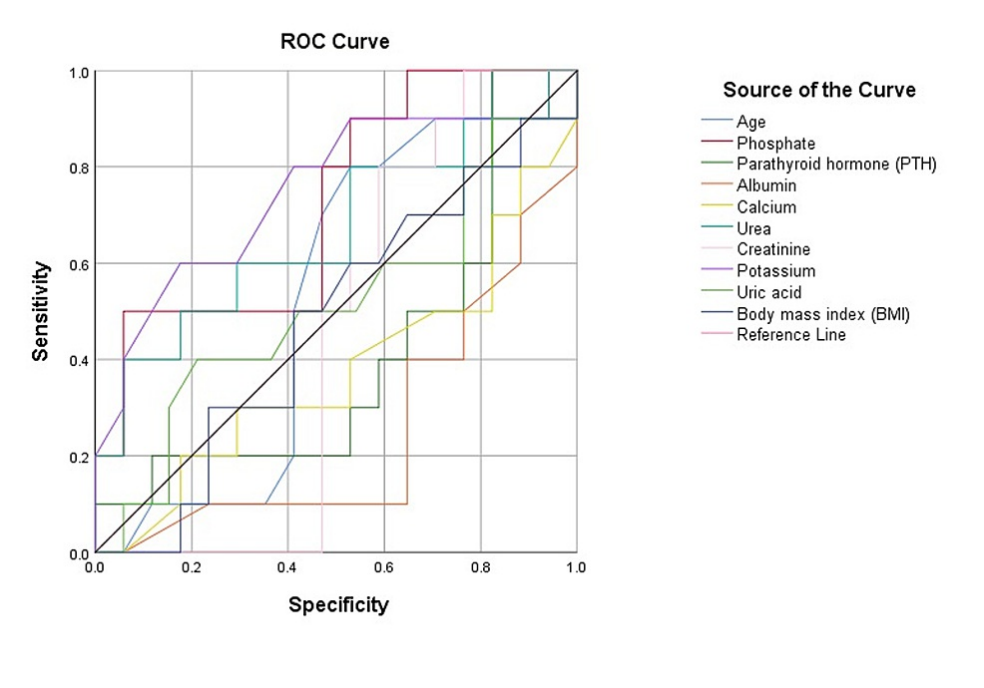
Combined ROC curves of all study variables. ROC: receiver operating characteristic; PTH: parathyroid hormone; BMI: body mass index.

Figure 3 shows combined ROC curves for all study variables. 

## Discussion

Cutaneous manifestation frequently associated with end-stage renal disease is pruritus, and this symptom results in significant changes in the quality of life of the sufferer, sleep disturbances, and depression potentiating the risk of morbidity [3]. The majority of patients belonged to an age range of 50-61 years [2,3,5,10,11]. No gender discrimination was observed in the development of pruritus among patients of CKD in multiple studies [3,5,11,12], while a few studies reported increased prevalence in the male gender [2,10]. Studies usually categorized the patients of end-stage renal disease in the presence and absence of pruritus [3,5,12], while a couple of studies categorized patients according to the severity of pruritus i.e., mild, moderate, and severe [2,10]. Abundant studies reported no statistically prominent difference in terms of age, sex, duration of dialysis, etiology of uremia, levels of PTH, calcium, phosphorus, and creatinine in association with pruritus among patients of end-stage renal disease [2,5,10]. Another study conducted by Massry et al. reported significantly raised levels of phosphorus manifesting pruritus [11]. A study of the same accord regulated in the Portuguese population reported prominent changes in pre-dialysis urea levels, calcium, and creatinine in a majority of patients while a change in phosphorus level was not found as significant [3]. A study conducted within the population of Saudi Arabia reported a decline in levels of calcium in patients with CKD leading to pruritus, while higher concentrations of PTH were observed prominently in the age group of 15-35 years [12]. Calcium-phosphorus product (Ca-P product) was found to increase in a study regulated by Massry et al. [11], while no prominent association of Ca-P product was detected with pruritus in the other study [12]. Serum alkaline phosphatase (ALP) levels were found to be elevated in pruritus suffering from CKD patients in various studies [5,11]. Frequent comorbidity encountered among patients was diabetes mellitus [2,5]. Massry et al. reported an increased prevalence of bone disease and enlarged parathyroid along with hyperparathyroidism in patients with pruritus [11], while another study reported symptoms of neuropathy prominent in patients of chronic kidney disease with pruritus [5]. Akhyani and colleagues reported around 70% of their patients with generalized pruritus prominent at trunk and limbs with 31.4% of them developing it during or after dialysis [5].

Conventionally, pruritus is known to occur more with peritoneal dialysis [13]; however, no significant difference was reported in another study [14]. Multiple factors were reported to be associated with uremic pruritus, like inadequate dialysis, hyperparathyroidism, elevated Ca-P product, xerosis, raised serum magnesium, and aluminum concentrations [15]. Other less significant associations are anemia, vitamin-A, beta-2 microglobulin levels [16], human leukocyte antigen (HLA) B35, congestive cardiac failure, neurological disorder, and ascites. In our study, we demonstrated a significant association with hyperkalemia, hyperphosphatemia, hypoalbuminemia, female gender, and previous history of skin allergy. No significant associations were found with hypocalcemia, hyperparathyroidism, serum urea or creatinine, body mass index, diabetes, hypertension, connective tissue disorder, and frequency of dialysis. Our study findings of hyperphosphatemia were supported by Narita et al. [2], with an OR of 1.7 at a cut-off value of 6.6 mg/dL, however, our cut-off was 4.5 mg/dL with OR of 3.8 and an AUC of 0.724, sensitivity of 90% and PPV of 88.8%. The other findings of their study were contradicting with our results such as blood urea nitrogen >81 mg/dL with OR 1.4 and calcium >9.5 mg/dL with OR 1.4 were more associated, while PTH <200 pg/mL with OR 0.6 was less likely to be associated with pruritus [2]. Male gender was suffering more in their study while we report a significant association of female gender in our findings [2]. Another study reported a much higher prevalence of pruritus than our study (48% vs 37%) with significant associations including onset coincident with starting dialysis, the persistence of symptoms, elevated phosphate, parathyroid hormone (PTH), and blood urea nitrogen (BUN) level. The same study suggested elevated calcium associated with pruritus, which contradicted our findings of hypocalcemia, although statistically insignificant [17]. The frequency of pruritus reported by them was 46% on a daily basis and 52% as weekly or monthly, while we reported occurrence of pruritus on daily basis in 13.3%, weekly in 7.4%, monthly in 4.4%, and multiple times a day in 11.9% of the participants. The use of angiotensin-converting enzyme inhibitors (ACEI) was also associated with pruritus as reported by Zucker et al. [17], while loop diuretics were not found significant.

The management of pruritus in CKD includes optimum dialysis frequency, optimum treatment of hyperparathyroidism [11], hyperphosphatemia [18], and hypermagnesemia [15], regular use of emollients (like calamine lotion was reported in our study with a non-significant association), improving dialysis efficacy, topical analgesics (open-label use of the glycerol and paraffin containing emulsion caused substantial improvement of pruritus and quality of life of the hemodialysis patients in one study) [19], oral antihistaminics [20], montelukast [21], gabapentin/pregabalin [22], and antidepressants [23]. In our study, we tried to address two of the most followed therapies prevalent in our population i.e, topical steroids and topical calamine lotion although both were found insignificantly associated with pruritus, however, known skin allergy was another common phenomenon present in our study in 3.04% individuals (with or without pruritus) and was found single most important and independent factor for pruritis followed by laboratory parameters. Therefore, pruritus is reported to be caused by multiple factors hence affects the quality of life, and is also associated with poor outcomes in chronic hemodialysis patients [2,4,15,24].

There were a few limitations of our study, as it was a single dialysis center assessment, the sample size was limited due to participants with multiple exclusion criteria like chronic liver disease (CLD), inadequate/non-compliance to the frequency of dialysis, and known use of therapies other than calamine/topical steroids. However, multiple factors were missing from analysis like anemia status, liver function enzymes especially alkaline phosphatase, and serum magnesium among others. Still, the study significantly correlated many factors with the occurrence of pruritus in hemodialysis patients, most likely were history of skin allergy and serum phosphate levels followed by others.

## Conclusions

There is limited data suggestive of treating hyperparathyroidism, hyperphosphatemia, and elevated Ca-P product actually reduces uremic pruritus. However, many observational studies have been linking these factors independently to pruritus in hemodialysis patients. Apart from topical treatments, controlled studies must be conducted on the efficacy of different therapeutic regimens for the control of pruritus in CKD patients as it is associated with poor quality of life and mortality.
